# Investigation and Correlation Analysis of Pathogens Carried by Ticks and Cattle in Tumen River Basin, China

**DOI:** 10.3390/vetsci13010078

**Published:** 2026-01-13

**Authors:** Pengfei Min, Jianchen Song, Yinbiao Meng, Shaowei Zhao, Zeyu Tang, Zhenyu Wang, Sicheng Lin, Fanglin Zhao, Meng Liu, Longsheng Wang, Lijun Jia

**Affiliations:** 1 Engineering Research Center of North-East Cold Region Beef Cattle Science & Technology Innovation, Ministry of Education, Yanbian University, Yanji 133002, China; 2 State Key Laboratory for Diagnosis and Treatment of Severe Zoonotic Infectious Diseases, Key Laboratory for Zoonosis Research of the Ministry of Education, College of Veterinary Medicine, Jilin University, Changchun 130062, China

**Keywords:** Tumen River Basin, ticks, cattle, pathogens, epidemiology

## Abstract

Ticks and tick-borne diseases pose a significant threat to public health. The Tumen River Basin is located at the junction of China, North Korea and Russia, whose warm climate and favorable ecological environment are suitable for the growth and reproduction of ticks. At the same time, the cattle industry in this region is highly developed, with cattle serving as the primary economic source for the area. This study performed an epidemiological investigation and analysis of pathogens carried by ticks and cattle in the Tumen River basin. A total of 913 ticks and 247 bovine blood samples were collected from seven cities primarily focused on cattle farming in the Tumen River basin. Morphological and molecular biological identification of ticks was carried out to determine the distribution of ticks and their pathogens in the region. Through the detection of pathogens carried by cattle blood samples in the surrounding area, the correlation with tick distribution was confirmed. The species and distribution of ticks of different genders and in different collection environments, and the infection of pathogens in bovine blood samples were statistically analyzed. The results showed that the 913 ticks had 5 species, including *Haemaphysalis longicornis*, *Haemaphysalis concinna*, *Haemaphysalis japonica*, *Dermacentor silvarum* and *Ixodes persulcatus*. Three pathogens, *Babesia ovata*, *Theileria orientalis* and *Theileria sinensis*, were detected in the blood samples of vector ticks and cattle. These results provide a theoretical basis for the prevention and control of ticks and tick-borne diseases in the Tumen River basin.

## Simple Summary

Ticks are a significant external parasite of livestock, capable of transmitting diseases to animals through blood-feeding, resulting in substantial economic losses to the livestock industry. The Tumen River is located in the northeastern border region of China, and there is little information about which tick species infest cattle and which can carry harmful blood parasites. This study was conducted in seven regions of the Tumen River Basin. There are 5 species of ticks in the Tumen River basin including *Haemaphysalis longicornis*, *Haemaphysalis concinna*, *Haemaphysalis japonica*, *Dermacentor silvarum* and *Ixodes persulcatus*. Three pathogens posing significant threats to livestock, particularly cattle, were detected in blood samples from ticks and cattle: *Babesia ovata*, *Theileria orientalis*, and *Theileria sinensis*. The pathogens detected in tick and cow blood are highly homologous. These findings indicate that ticks in the Tumen River basin may serve as vectors for *B. ovata*, *T. orientalis*, and *T. sinensis*. Cattle populations within the basin are susceptible to contracting these three diseases through tick bites. Additionally, these ticks pose a potential threat to public health. Although there is currently insufficient evidence to indicate that humans can be infected by the above three pathogens, ticks as the primary vectors for other zoonotic pathogens—such as *Borrelia burgdorferi*—should be taken seriously, necessitating heightened vigilance and control measures by farmers and livestock breeders. These results improve knowledge of tick species in the border region of China and show the importance of continued monitoring to protect animal health and farming.

## 1. Introduction

The tick is a blood-sucking arthropod found on every land on Earth and is parasitic on most vertebrates, such as humans, mammals, reptiles and birds [1,2]. There are records of tick infestation as early as 1550 BC [3]. The survival time of ticks is generally two years. Except for individual ticks, all four stages of tick growth require blood. Ixodes takes a blood meal once at different stages of growth, while soft ticks can take blood meals multiple times [4,5]. In addition to the physical bite of the tick, which can make people and animals feel pain and develop allergic reactions, wasting, poisoning and other adverse effects, the most harmful is the vector role of the tick [2]. Ticks can transmit pathogens such as protozoa, viruses and bacteria as they feed [6]. Tick-borne pathogens need to be acquired from susceptible hosts and passed on to new hosts as they move to the next stage of growth, so that tick-borne pathogens remain. The number of zoonotic pathogens transmitted by ticks as vectors is the largest and increasing [7,8,9]. This directly damages human and animal health and indirectly affects the economy and development of society. Ticks are most common in tropical and subtropical regions, and therefore cause the greatest economic damage in these regions [10]. With the expansion of tick distribution and tick-borne disease epidemic areas, more effective control methods and risk monitoring are urgently needed. In recent years, various newly discovered tick-borne pathogens have brought great challenges to the prevention and control of tick-borne diseases.

*Piroplasmida* is a genus of protozoan parasites that infect the red blood cells and lymphocytes of vertebrates, primarily transmitted by ticks. It can infect domestic animals such as horses, cattle, sheep, and camels, posing the greatest threat to cattle. *Babesia* and *Theileria* are the two main families of *Piroplasmida* [11]. *Babesia* is divided into numerous subspecies, including *Babesia caballi*, *Babesia ovata*, *Babesia bigemina* and others [12]. The *Theileria* is primarily divided into three subtypes: *Theileria sergenti*, *Theileria orientalis* and *Theileria sinensis* [13]. The Tumen River Basin, as a major livestock breeding base in Northeast China, has seen limited research on the three pathogens *Babesia ovata*, *Theileria orientalis* and *Theileria sinensis*, despite their significant harm to cattle.

The Tumen River basin is located at the junction of China, North Korea and Russia, with high forest coverage, rich vegetation, a wide variety of wild animals and relatively developed animal husbandry [14]. The basin’s warm climate and favorable ecological environment are suitable for tick growth and reproduction. The Tumen River Basin is an important ecological functional area, but the species of ticks and the prevalence of tick-borne diseases in the basin are still unclear. Therefore, it is necessary to carry out investigation and research to better understand the tripartite relationship between ticks, tick-borne diseases and animal hosts. This study provides a scientific basis for the prevention and control of ticks and tick-borne diseases in the Tumen River Basin, and provides guidance for the treatment of tick-borne diseases in the basin.

## 2. Materials and Methods

### 2.1. Sample Collection

**Figure 1:**
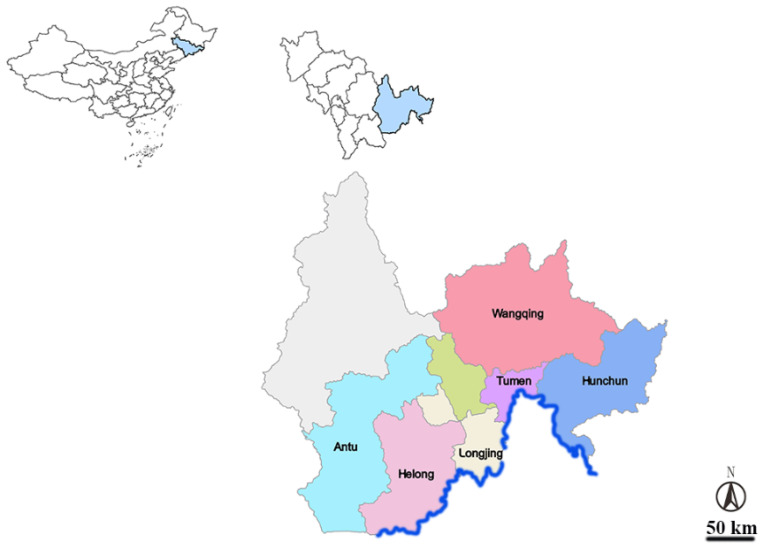
Map of sampling districts in Tumen River Basin, China. The different colors represent the various districts sampled in this study. The blue line represents the Tumen River.

From March 2021 to October 2022, ticks living free in forest and grassland and ticks living on the body surface of cattle were collected in seven counties and cities primarily focused on cattle farming around the Tumen River basin, including Antu, Helong, Hunchun, Longjing, Tumen, Wangqing and Yanji (Figure 1). Free ticks were collected using the cloth flag method. Ticks on the bovine body surface are collected using tweezers. EDTA-containing bovine venous blood samples were collected from cattle ranches and cattle farmers in the tick collection areas of Antu, Hunchun, Helong, Tumen and Yanji. Cattle are classified by sex into male and female, by management into graze and farm, and by age into three stages: <1 years old; 1–2 years old; >2 years old. The sampling season corresponds to the period of peak tick activity in the region—spring. The months involved are April through June each year. Samples are collected once per month, totaling six collections over a two-year period. Ticks and bovine blood samples are collected on the same day. Ticks were classified according to location, time, breed, environment, sex and other information. A total of 913 ticks and 247 bovine blood samples were collected.

### 2.2. Tick Classification and Nucleic Acid Extraction

The morphological structures of different species of male and female ticks were observed with a stereomicroscope. Each tick was washed three times in normal saline solution and placed in a 1.5 mL centrifuge tube, and 600 μL phosphate-buffered saline was added. The ticks were then ground with a tissue breaker, followed by centrifugation at 1300× *g* for 1 min. Approximately 200 μL of the supernatant was collected to extract the nucleic acid. The genomic DNA from the tick was extracted using the TIANamp Genomic DNA kit (TIANGEN, Beijing, China). DNA should be stored at −20 °C.

### 2.3. Bovine Blood Nucleic Acid Extractio

The genomic DNA from the bovine blood was extracted using the TIANamp Blood DNA Kit (TIANGEN, Beijing, China). DNA should be stored at −20 °C.

### 2.4. Detection of Pathogens in Ticks and Bovine Blood

Polymerase Chain Reaction (PCR) was used to detect pathogens in DNA extracted from ticks and bovine blood. Species-specific primers were used to amplify the chaperonin-containing t-complex polypeptide **1** (*CCTeta*) gene of *Babesia ovata* and the major piroplasm surface protein (*MPSP*) gene of *Theileria sinensis* and *Theileria orientalis*. The primers used in this study are listed in Table 1. The PCR was conducted with a 25 µL reaction volume comprising 1 µL of reverse and forward primer (10 pmol), 2 µL of template DNA (60–80 ng/µL), 12.5 µL of Green Taq Mix (Vazyme, Nanjing, China) and 10.5 µL of distilled water. The PCR conditions are presented in Table 2.

### 2.5. Sequence Identity and Phylogenetic Analyses

The PCR products of the positive samples were sent to Shanghai Shenggong Biotechnology Company for Sanger sequencing. Newly obtained sequences were compared with the National Center for Biotechnology Information database (https://www.ncbi.nlm.nih.gov/, accessed on 15 May 2023) using the Basic Local Alignment Search Tool (https://blast.ncbi.nlm.nih.gov/Blast.cgi, accessed on 15 May 2023) and related sequences were retrieved from the GenBank database (https://www.ncbi.nlm.nih.gov/genbank/, accessed on 15 May 2023). ClustalW software (https://clustalw.com/, accessed on 20 May 2023) was used for multiple sequence alignment. Phylogenetic trees were constructed using the Neighbor-Joining method with a *p*-distance model and bootstrapping of 1000 replicates to calculate the evolutionary relationship using Molecular Evolutionary Genetics Analysis software (v.11.0; https://www.megasoftware.net/, accessed on 6 June 2023)

### 2.6. Statistical Analysis

The chi-squared test was used to compare proportions of detected sample positivity in different condition via Prism 9 software (GraphPad Software, LLC, San Diego, CA, USA). When the *p*-value was lower than 0.05, the difference was considered statistically significant.

## 3. Results

### 3.1. Tick Species Survey Results

**Figure 2:**
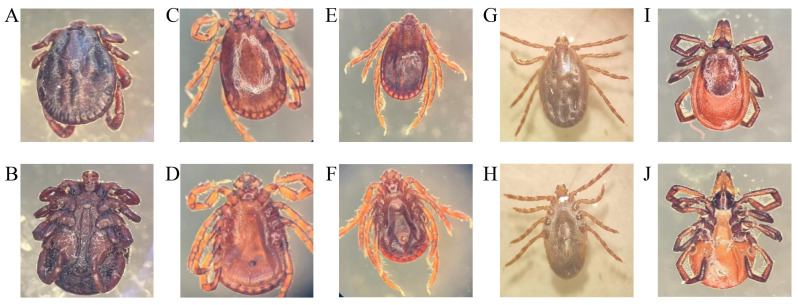
Morphological identification of ticks. (**A**,**B**) *Dermacentor silvarum*; (**C**,**D**) *Haemaphysalis concinna*; (**E**,**F**) *Haemaphysalis japonica*; (**G**,**H**) *Haemaphysalis Iongicornis*; **(I**,**J**) *Ixodes persulcatus*.

According to the identification results, there were three genera and five species of ticks in Tumen River basin, which included 148 *Haemaphysalis longicornis*, 152 *Haemaphysalis japonica*, 164 *Haemaphysalis concinna*, 39 *Ixodes persulcatus*, and 410 *Dermacentor silvarum* with proportions of 16.21%, 16.65%, 17.96%, 4.27%, and 44.91% (Figure 2A–J and Table 3).

### 3.2. Results of Pathogen Investigation

We detected *Babesia ovata* in *H. longicornis*, *I. persulcatus*, *H. japonica* and *H. concinna*; *Theileria orientalis* in *H. longicornis*, *H. japonica* and *H. concinna;* and *Theileria sinensis* in *H. longicornis* and *H. japonica*. In terms of age, adult ticks are more likely to carry pathogens than nymphs. In different environments, the detection rate of *T. sinensis* on the body surface was significantly increased, the detection rate of *T. orientalis* on the body surface of animals was lower than that of forest and grassland, and the detection rate of *B. ovata* was not significantly changed in the three environments (Table 4). All three pathogens were detected in bovine blood. Age and sex had significant effects on the infection rate of *T. orientalis* in cattle, but feeding style did not affect the infection rate. The opposite is true for *T. sinensis* and *B. ovata* (Table 5).

### 3.3. Phylogenetic Analysis of Pathogen

**Figure 3:**
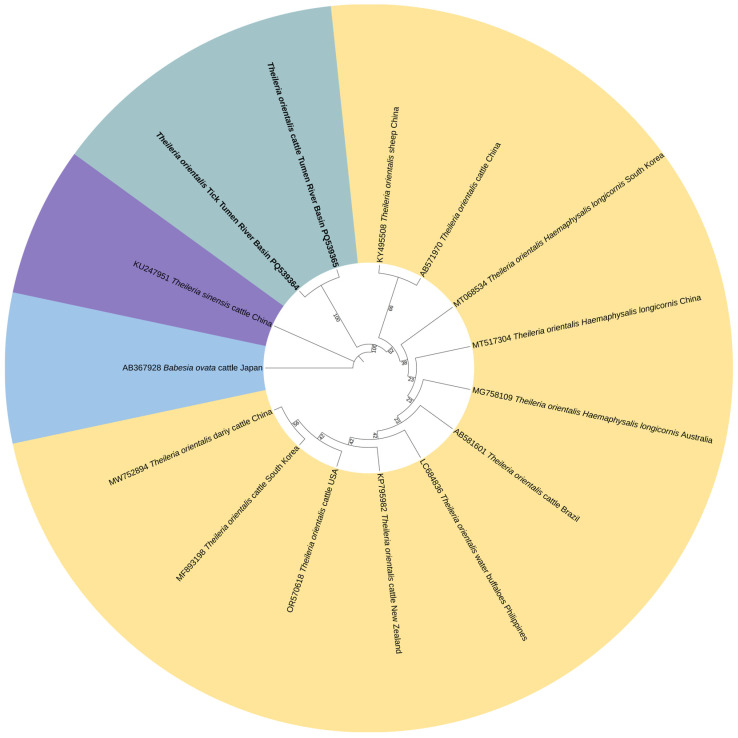
ML-based phylogenetic analysis of *MPSP* gene sequences of the *T. orientalis* isolate. Tamura 3-parameter model with 1000 bootstrap replications. The sequences obtained in this study are in black bold text. The phylogenetic tree was visualized using ITOL software (version v7).

**Figure 4:**
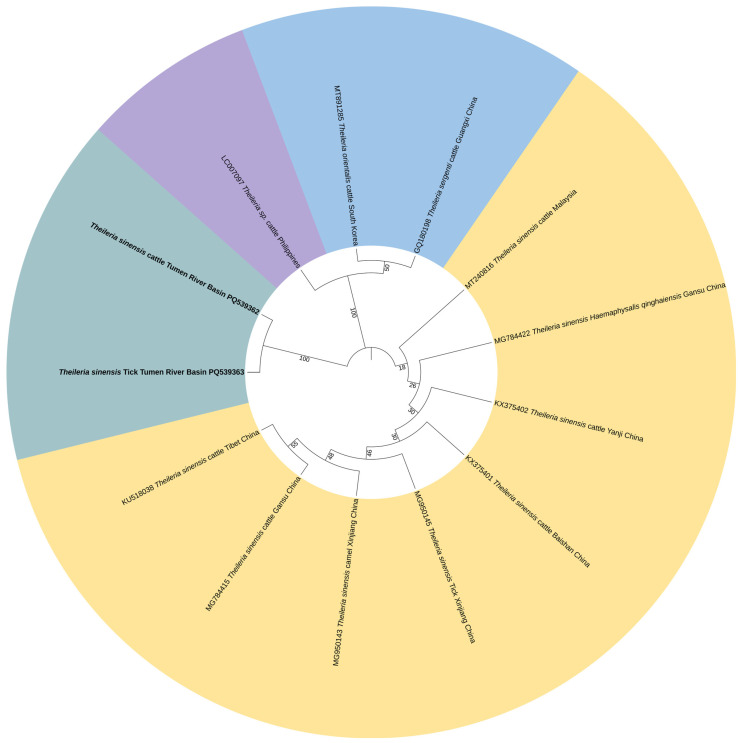
ML-based phylogenetic analysis of *MPSP* gene sequences of the *T. sinensis* isolate. Tamura 3-parameter model with 1000 bootstrap replications. The sequences obtained in this study are in black bold text. The phylogenetic tree was visualized using ITOL software.

**Figure 5:**
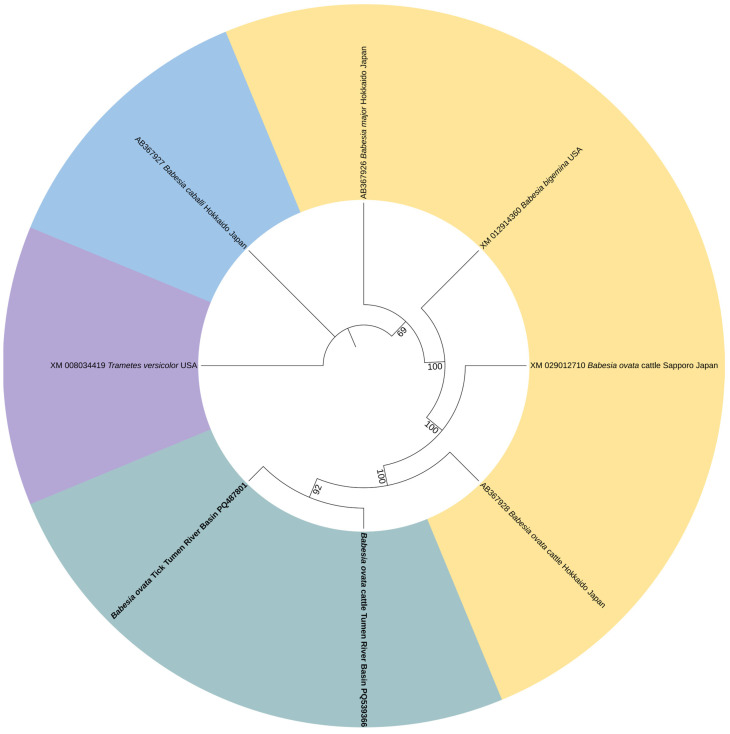
ML-based phylogenetic analysis of *CCTeta* gene sequences of the *B. ovata* isolate. Tamura 3-parameter model with 1000 bootstrap replications. The sequences obtained in this study are in black bold text. The phylogenetic tree was visualized using ITOL software.

Phylogenetic analysis showed that the *T. orientalis MPSP* gene sequences generated from ticks (PQ539364) and cattle (PQ539365) in the Tumen River Basin clustered with the South Korea strain (MF893198), Brazil strain (AB581601), and Australia strain (MG758109) (Figure 3). In a similar manner, the *T. sinensis MPSP* gene sequences generated from ticks (PQ539363) and cattle (PQ539362) obtained in this study shared 100% identities and clustered with sequences from Gansu China (MG784422), Baishan China (KX375401), and Malaysia (MT240816) (Figure 4). The nucleotide sequence identity data demonstrated that sequences of the *B. ovata CCTeta* gene obtained in this study shared more than 95% identity with most of the *B. ovata CCTeta* gene sequences previously identified in Japan. The phylogenetic analysis showed the *B. ovata CCTeta* gene sequence from ticks (PQ487801) and cattle (PQ539366) in the Tumen River Basin in the same clade as the *B. ovata CCTeta* gene sequence (AB367928) from Hokkaido cattle in Japan (Figure 5).

## 4. Discussion

Tumen River Basin is located at 41°59′~44°1′ North latitude, 128°17′–131°18′ East longitude, with a total drainage area of 33,168 square kilometers. This basin is located in the main area of Changbai Mountain, affected by the topographic height, and the climate elements have vertical changes. The Tumen River Basin serves as a vital ecological functional zone, featuring diverse wetland types, fertile soils, and abundant water resources. The mild climate and rich biodiversity in the Tumen River Basin are conducive to tick breeding. Therefore, it is of great significance to investigate the species and distribution of ticks in the Tumen River Basin for the prevention and control of tick-borne diseases. In this study, 913 ticks collected from seven counties and cities in the Tumen River Basin were classified and analyzed. Among the five identified species, *D. silvarum* was the dominant tick species in the Tumen River Basin.

*B. ovata* mainly parasitizes the red blood cells of cattle [15]. It is a kind of large piroplasms; the piroplasms is round, oval and pear seed in shape; each body contains 1~2 clusters of chromatin; and the central cavity is often formed [18]. *B. ovata* produces asexually in the red blood cells of the intermediate host, such as cattle and sheep, forming two or four bodies by way of budding [19]. Chinese scholars have also found *B. ovata* in ticks collected from the body surface of naturally infected cattle [20]. The positive rate of *B. ovata* among 913 ticks in the Tumen River Basin was 5.36%. All counties and cities had different degrees of infection. According to regional distribution, the infection rate of *B. ovata* in the middle reaches of the Tumen River Basin was the highest. *B. ovata* was detected in *H. longicornis*, *H. concinna*, *H. japonica* and *I. persulcatus*. The infection rate of *H. longicornis* was the highest as the main transmission vector. There was no obvious change trend in *B. ovata* in different environments. According to the investigation results, *B. ovata* was detected in tick and bovine blood samples in the upper, middle and lower reaches of the Tumen River basin, indicating that the Tumen River basin is an endemic area of *B. ovata*. The positive rate in ticks was significantly lower than that in bovine blood samples. In addition, *B. ovata* was detected in the blood samples and ticks collected in the same county and city, and the sequence analysis homology was 100%, indicating that there is a certain correlation between *B. ovata* infection in cattle and ticks. Attention should be paid to preventing tick transmission of *B. ovata* in the Tumen River basin. *B. ovata* poses the most severe threat to cattle. Currently, there is insufficient evidence to indicate that humans can contract *B. ovata* through tick bites. However, this does not mean we can lower our guard. For farmers and livestock breeders in particular, preventing cattle from being bitten by ticks remains a key concern that requires their attention.

*T. orientalis* is very harmful to cattle and can kill cattle in severe cases [21]. It is mainly transmitted by ticks, and cases of transmission of *T. orientalis* by the invasive *H. longicornis* have been reported in Virginia, USA [22]. The disease is found all over the world, with New Zealand, Australia and Japan among the endemic countries [23]. In this study, the positive rate of *T. orientalis* detected in ticks was 12.04%, and seven counties and cities were infected. This indicates that *T. orientalis* is widely distributed in the Tumen River basin. According to the regional distribution characteristics, the positive rate in the middle reaches of the Tumen River basin was the highest, and the positive rate in the upper reaches was significantly lower than that in the middle and lower reaches. *H. longicornis*, *H. concinna, H. japonica* and *T. orientalis* were detected. The detection rate of *H. longicornis* was the highest. These results indicate that different species of ticks have great differences in the transmission of *T. orientalis*. *T. orientalis* was detected in the blood of ticks and cattle in all counties and cities of the Tumen River basin. The sequence homology analysis of ticks and cattle showed 98.7%, indicating that cattle infection with *T. orientalis* was related to tick transmission.

*T. sinensis* was first discovered in Gansu, China, and *Haemaphysalis Qinghaiensis* and *H. japonica* have the ability to transmit *T. sinensis* [24,25]. *T. sinensis* is currently considered to be a novel tick-borne pathogen. It has been reported that the existence of *T. sinensis* has been confirmed in yaks in Qinghai for the first time [26]. *T. sinensis* was detected in *H. longicornis* and *H. japonica* in Hunchun, which may be related to environmental differences between regions. This survey is the first time that *T. sinensis* in *H. japonica* and *H. longicornis* was found in the Tumen River basin, and, especially, the discovery of *T. sinensis* in *H. japonica* should be paid attention to. The homology of tick and bovine origin of *T. sinensis* was 100%, indicating that *T. sinensis* prevalent in the Tumen River basin was of the same origin, but the positive rate of *T. sinensis* in ticks was only 0.87%. Whether the main cause of *T. sinensis* infection in cattle was tick transmission remains to be further studied.

The prevention and control of ticks and tick-borne diseases remain a serious challenge. For most farmers and livestock breeders, scientifically effective tick prevention remains the key to interrupting the transmission of tick-borne diseases. Currently, classic topical medications such as permethrins play a vital role in preventing tick infestations. Surveys indicate that treating clothing with permethrin is highly effective against ticks. It can reduce the probability of being bitten by ticks by 93% [27]. In animal studies, permethrins also demonstrated good preventive and curative effects [28]. Treating farmers’ and livestock breeders’ clothing with pesticides is a simple and effective method for tick control. This approach is not only effective against ticks but also useful for controlling insects such as mosquitoes [29]. However, each method has certain limitations. Reports indicate that while traditional acaricides may yield some results in small-scale experiments, their effectiveness significantly diminishes as the scope of research expands [30]. Additional studies indicate that employing the classic “4-Posters” method can significantly reduce tick populations within the study area [31]. These also proved, although effective, not ‘cost-effective’ given the tons of corn required to attract deer. In summary, there are ‘strategic’ and practical measures that the farmers or public health infrastructures can adopt to reduce the scourge of ticks and tick-vectored diseases. This is a key focus area for our future research.

The primary method employed in our study was PCR, a highly specific detection technique. Of course, our research has certain limitations. While PCR is the most common method for detecting tick-borne pathogens, it can only detect a single pathogen at a time, making it prone to overlooking co-infections [32]. With technological advancements, Next-Generation Sequencing (NGS) has gained widespread application in pathogen detection. This technology can identify tens of millions of sequences within a sample and, through bioinformatics analysis, determine all potential pathogens present in the sample [33]. NGS has a significantly enhanced detection efficiency and is likely to become a hotspot for future research. Additionally, this study did not perform serological testing to determine which tick-borne pathogens elicited immune responses in the animals. This work will be conducted in our subsequent research. As is well known, ticks can carry a wide variety of pathogens, with *Babesia* alone exhibiting numerous genotypes [34]. However, due to limited research funding, we selected only a few representative pathogens for illustration in this study. We plan to conduct in-depth research and discussion on the gaps identified in this investigation in our next phase.

## 5. Conclusions

There are 5 species of ticks in the Tumen River basin. Three pathogens *Babesia ovata*, *Theileria orientalis* and *Theileria sinensis* were detected in ticks and cattle blood in Tumen River basin. The pathogens detected in tick and cow blood are highly homologous. This study suggests that ticks may be the transmission vector of the above three pathogens, and farmers should focus on prevention. Our research provides theoretical support for future disease prevention and control in the Tumen River Basin and enables precise prevention and control of imported risks through early warning systems. This offers theoretical support for promoting the sustainable development of animal husbandry and fostering harmonious coexistence between humans and nature.

## Figures and Tables

**Table 1:** Primer sequences used for the gene amplification of different pathogens.

Pathogen Gene	Primer Name	Sequence (5′–3′)	Fragment Size (bp)	Reference
*B. ovata CCTeta*	P1	GCCCGCAGGTCATCATAAAGT	1008	[15]
	P2	CATTTTGTGCCAGCGTTTTG		
*T. orientalis MPSP*	P3	CCAAGTTCACCCCAACTGTCG	499	[16]
	P4	GCACTGTTCATGGCGTGCAAA		
*T. sinensis MPSP*	P5	CACTGCTATGTTGTCCAAGAGATATT	887	[17]
	P6	AATGCGCCTAAAGATAGTAGAAAAC		

**Table 2:** Reaction conditions of various primers.

Pathogen Gene	Pre-Denaturation Temperature (°C) /Time (s)	Denaturation Temperature (°C) /Time (s)	Annealing Temperature (°C) /Time (s)	Stretching Temperature (°C) /Time (s)	Temperature of Re-Extension (°C) /Time (s)	Cycle	Storage Temperature (°C) /Time (s)
*B. ovata CCTeta*	95/300	95/30	51.4/30	72/30	72/480	35	4
*T. orientalis MPSP*	94/300	94/60	58/60	72/60	72/420	35	4
*T. sinensis MPSP*	94/300	94/60	56/60	72/60	72/420	35	4

**Table 3:** Composition of tick species in seven counties and cities of Tumen River Basin.

Location	*Haemaphysalis* *longicornis*		*Ixodes* *persulcatus*		*Haemaphysalis* *japonica*		*Dermacentor* *silvarum*		*Haemaphysalis* *concinna*		Total	
	Quantity	Constituent Ratio (%)	Quantity	Constituent Ratio (%)	Quantity	Constituent Ratio (%)	Quantity	Constituent Ratio (%)	Quantity	Constituent Ratio (%)	Quantity	Constituent Ratio (%)
Hunchun	136	55.97	7	2.88	8	3.29	63	25.93	29	11.93	243	100.00
Tumen	0	0.00	0	0.00	2	3.39	47	79.66	10	16.95	59	100.00
Yanji	0	0.00	0	0.00	33	15.00	165	75.00	22	10.00	220	100.00
Helong	0	0.00	0	0.00	19	76.00	0	0.00	6	24.00	25	100.00
Longjing	0	0.00	6	3.11	31	16.06	59	30.57	97	50.26	193	100.00
Antu	12	8.45	20	14.09	34	23.94	76	53.52	0	0.00	142	100.00
Wangqing	0	0.00	6	19.35	25	80.65	0	0.00	0	0.00	31	100.00
Total	148	16.21	39	4.27	152	16.65	410	44.91	164	17.96	913	100.00

**Table 4:** Pathogens statistics from ticks.

Variable	*Babesia ovata* Positive Rate (%)	*Theileria orientalis* Positive Rate (%)	*Theileria sinensis* Positive Rate (%)
Region			
Hunchun	11.11	25.11	2.88
Tumen	3.39	1.69	0.00
Yanji	2.72	5.45	0.00
Helong	8.00	28.00	4.00
Longjing	3.10	3.62	0.00
Antu	2.81	9.85	0.00
Wangqing	19.35	20.58	0.00
Type			
*Haemaphysalis longicornis*	18.91	42.56	2.70
*Ixodes persulcatus*	5.10	0.00	0.00
*Haemaphysalis japonica*	11.25	12.12	2.64
*Dermacentor silvarum*	0.00	0.00	0.00
*Haemaphysalis concinna*	1.21	11.51	0.00
Sex			
Male	5.83	13.70	0.00
Female	6.40	11.60	1.37
Nymph	0.00	6.50	0.16
Ambient			
Forest	5.10	11.60	0.00
Grass	6.41	13.27	0.88
Body surface	5.70	4.20	5.71

**Table 5:** Pathogens statistics from cattle.

Variable	*Babesia ovata* Positive Rate (%)	*Theileria orientalis* Positive Rate (%)	*Theileria sinensis* Positive Rate (%)
Region			
Hunchun	26.75	54.65	31.39
Yanji	0.00	10.81	18.92
Helong	7.89	31.57	63.15
Longjing	38.09	16.67	40.47
Antu	45.45	38.63	20.45
Sex			
Male	24.49	38.46	32.87
Female	25.96	30.77	35.58
Management			
Graze	27.38	35.71	36.9
Farm	20.25	34.18	27.85
Age			
<1 years old	26.92	40.38	32.69
1–2 years old	25.00	31.67	35.83
>2 years old	24.00	37.33	32

## Data Availability

The original data presented in the study are openly available in GenBank database with the accession numbers PQ487801 PQ539362–PQ539366.
